# Supplementary material for: TRIM6: An Upregulated Biomarker with Prognostic Significance and Immune Correlations in Gliomas
Source: Biomolecules. 2023 Aug 24;13(9):1298. doi: 10.3390/biom13091298 (PMC10527026; doi:10.3390/biom13091298)
Supplement: Supplementary file 1 [file biomolecules-13-01298-s001.zip › TableS1 Cox-Glioma_DSS.pdf]

Table S1 Cox regression analyses to explore the independent indicators of DSS in Gliomas

Characteristics	Total(N)	Univariate analysis		Multivariate analysis	
		Hazard ratio (95% CI)	P value	Hazard ratio (95% CI)	P value
WHO grade	614				
G2	220	Reference			
G3	239	3.098 (2.023-4.744)	**<0.001**	1.621 (0.983-2.674)	0.059
G4	155	19.243 (12.572-29.455)	**<0.001**	4.481 (1.370-14.650)	**0.013**
IDH status	664				
WT	232	Reference			
Mut	432	0.110 (0.083-0.146)	**<0.001**	0.468 (0.262-0.836)	**0.010**
1p/19q codeletion	668				
codel	169	Reference			
non-codel	499	4.987 (3.117-7.978)	**<0.001**	1.041 (0.510-2.124)	0.912
Primary therapy outcome	457				
PD	111	Reference			
SD	144	0.372 (0.242-0.572)	**<0.001**	0.312 (0.178-0.548)	**<0.001**
PR	64	0.138 (0.056-0.343)	**<0.001**	0.140 (0.043-0.457)	**0.001**
CR	138	0.116 (0.053-0.252)	**<0.001**	0.152 (0.067-0.348)	**<0.001**
Gender	674				
Female	289	Reference			
Male	385	1.248 (0.965-1.614)	0.092	1.711 (1.065-2.746)	**0.026**
Age	674				
<=60	541	Reference			
>60	133	4.500 (3.409-5.940)	**<0.001**	3.687 (2.146-6.332)	**<0.001**
Histological type	674				
Astrocytoma	192	Reference			
Glioblastoma	155	6.602 (4.739-9.197)	**<0.001**		
Oligoastrocytoma	132	0.604 (0.374-0.975)	**0.039**	1.107 (0.624-1.966)	0.728

Characteristics	Total(N)	Univariate analysis		Multivariate analysis	
		Hazard ratio (95% CI)	P value	Hazard ratio (95% CI)	P value
Oligodendroglioma	195	0.543 (0.363-0.813)	**0.003**	0.580 (0.321-1.046)	0.070
TRIM6	674				
Low	340	Reference			
High	334	4.421 (3.309-5.907)	**<0.001**	1.716 (1.080-2.726)	**0.022**

Characteristics: Variables and Groupings. **Total (N)**: Number of samples in each variable's total selected group and its respective subgroups. This represents the overall sample size for each variable and corresponding grouping, used for conducting univariate analysis. **HR (95% CI) Univariate analysis**: Hazard Ratio (HR) values obtained from the univariate analysis along with their corresponding confidence intervals (CIs). The "Reference" category represents the reference group for categorical variables, while other groups are compared to this reference group. **P value Univariate analysis**: p-value associated with the independent variable obtained from the univariate analysis. If it meets a predetermined threshold, it is considered significant and included in the multivariable model. **HR (95% CI) Multivariate analysis**: Only variables meeting the predefined p-value threshold (0.1) for inclusion in the multivariable Cox model will have values reported here. The histological type was not included in the multivariable regression analysis due to its collinearity with other variables. PD: progressive disease. SD: stable disease. PR: partial response. CR: complete response.
