# Supplementary material for: TRIM6: An Upregulated Biomarker with Prognostic Significance and Immune Correlations in Gliomas
Source: Biomolecules. 2023 Aug 24;13(9):1298. doi: 10.3390/biom13091298 (PMC10527026; doi:10.3390/biom13091298)
Supplement: Supplementary file 1 [file biomolecules-13-01298-s001.zip › TableS2 Cox-Glioma_PFI.pdf]

Table S2 Cox regression analyses to explore the independent indicators of PFI in Gliomas

Characteristics	Total(N)	Univariate analysis		Multivariate analysis	
		Hazard ratio (95% CI)	P value	Hazard ratio (95% CI)	P value
WHO grade	634				
G2	223	Reference			
G3	243	1.616 (1.197-2.182)	**0.002**	1.098 (0.771-1.565)	0.603
G4	168	7.865 (5.808-10.649)	**<0.001**	2.002 (0.674-5.946)	0.212
IDH status	685				
WT	246	Reference			
Mut	439	0.151 (0.119-0.191)	**<0.001**	0.412 (0.264-0.643)	**<0.001**
1p/19q codeletion	688				
codel	170	Reference			
non-codel	518	3.373 (2.438-4.666)	**<0.001**	1.195 (0.744-1.921)	0.460
Primary therapy outcome	461				
PD	112	Reference			
SD	147	0.253 (0.178-0.358)	**<0.001**	0.264 (0.177-0.394)	**<0.001**
PR	64	0.226 (0.137-0.372)	**<0.001**	0.233 (0.125-0.435)	**<0.001**
CR	138	0.160 (0.104-0.246)	**<0.001**	0.182 (0.113-0.295)	**<0.001**
Gender	695				
Female	297	Reference			
Male	398	1.083 (0.875-1.342)	0.463		
Age	695				
<=60	552	Reference			
>60	143	2.873 (2.268-3.640)	**<0.001**	2.122 (1.399-3.219)	**<0.001**
Histological type	695				
Astrocytoma	195	Reference			
Glioblastoma	168	4.416 (3.353-5.816)	**<0.001**		
Oligoastrocytoma	134	0.578 (0.401-0.832)	**0.003**	0.850 (0.550-1.314)	0.464

Characteristics	Total(N)	Univariate analysis		Multivariate analysis	
		Hazard ratio (95% CI)	P value	Hazard ratio (95% CI)	P value
Oligodendroglioma	198	0.638 (0.469-0.868)	**0.004**	0.677 (0.440-1.040)	0.075
TRIM6	695				
Low	347	Reference			
High	348	2.942 (2.354-3.676)	**<0.001**	1.090 (0.766-1.551)	0.633

Characteristics: Variables and Groupings. **Total (N)**: Number of samples in each variable's total selected group and its respective subgroups. This represents the overall sample size for each variable and corresponding grouping, used for conducting univariate analysis. **HR (95% CI) Univariate analysis**: Hazard Ratio (HR) values obtained from the univariate analysis along with their corresponding confidence intervals (CIs). The "Reference" category represents the reference group for categorical variables, while other groups are compared to this reference group. **P value Univariate analysis**: p-value associated with the independent variable obtained from the univariate analysis. If it meets a predetermined threshold, it is considered significant and included in the multivariable model. **HR (95% CI) Multivariate analysis**: Only variables meeting the predefined p-value threshold (0.1) for inclusion in the multivariable Cox model will have values reported here. The histological type was not included in the multivariable regression analysis due to its collinearity with other variables. PD: progressive disease. SD: stable disease. PR: partial response. CR: complete response.
