# Supplementary figures and images for: TRIM6: An Upregulated Biomarker with Prognostic Significance and Immune Correlations in Gliomas
Source: Biomolecules. 2023 Aug 24;13(9):1298. doi: 10.3390/biom13091298 (PMC10527026; doi:10.3390/biom13091298)

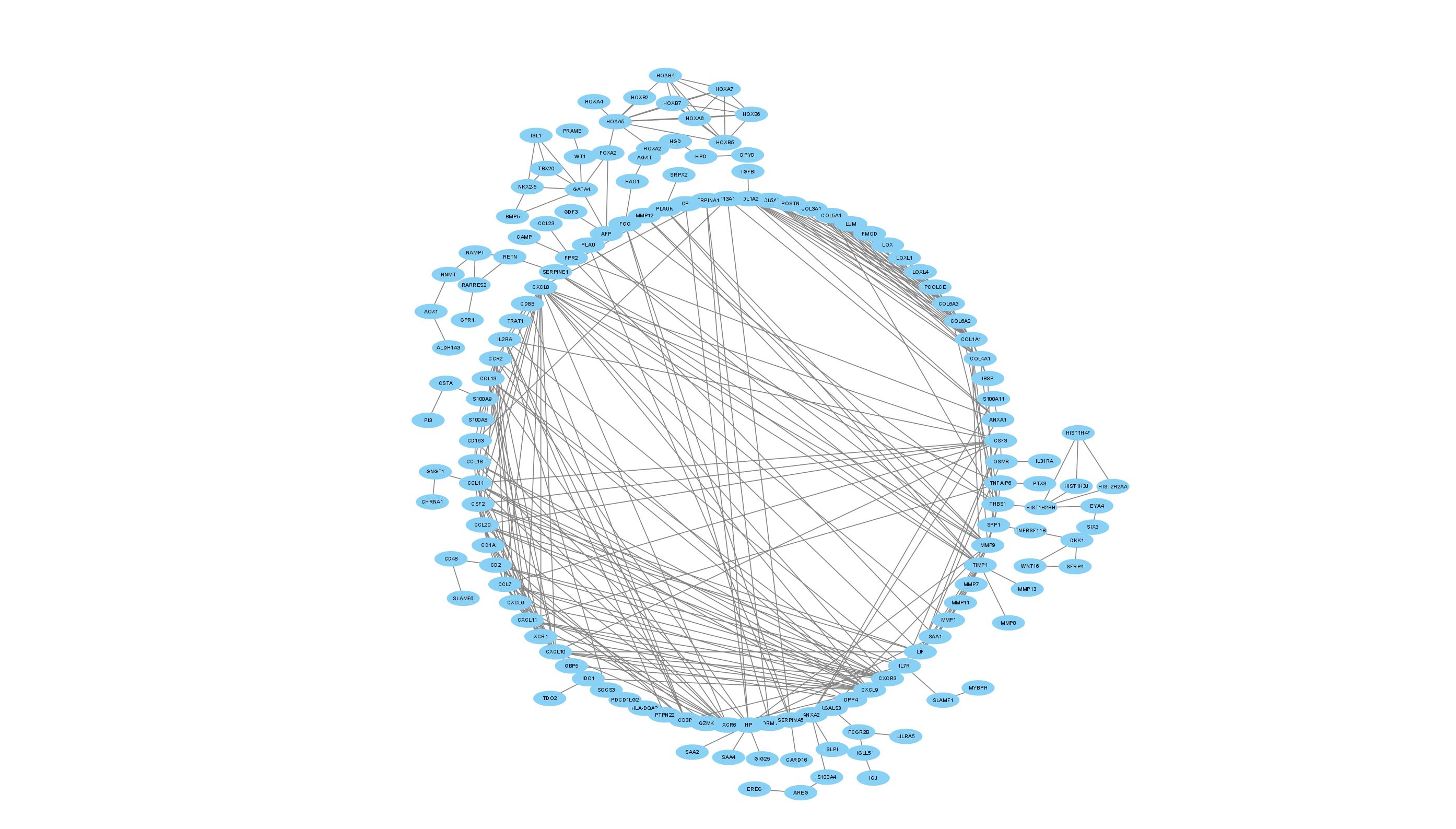

Supplement: Supplementary file 1 [file biomolecules-13-01298-s001.zip › HD_Figs. 7A.jpeg]

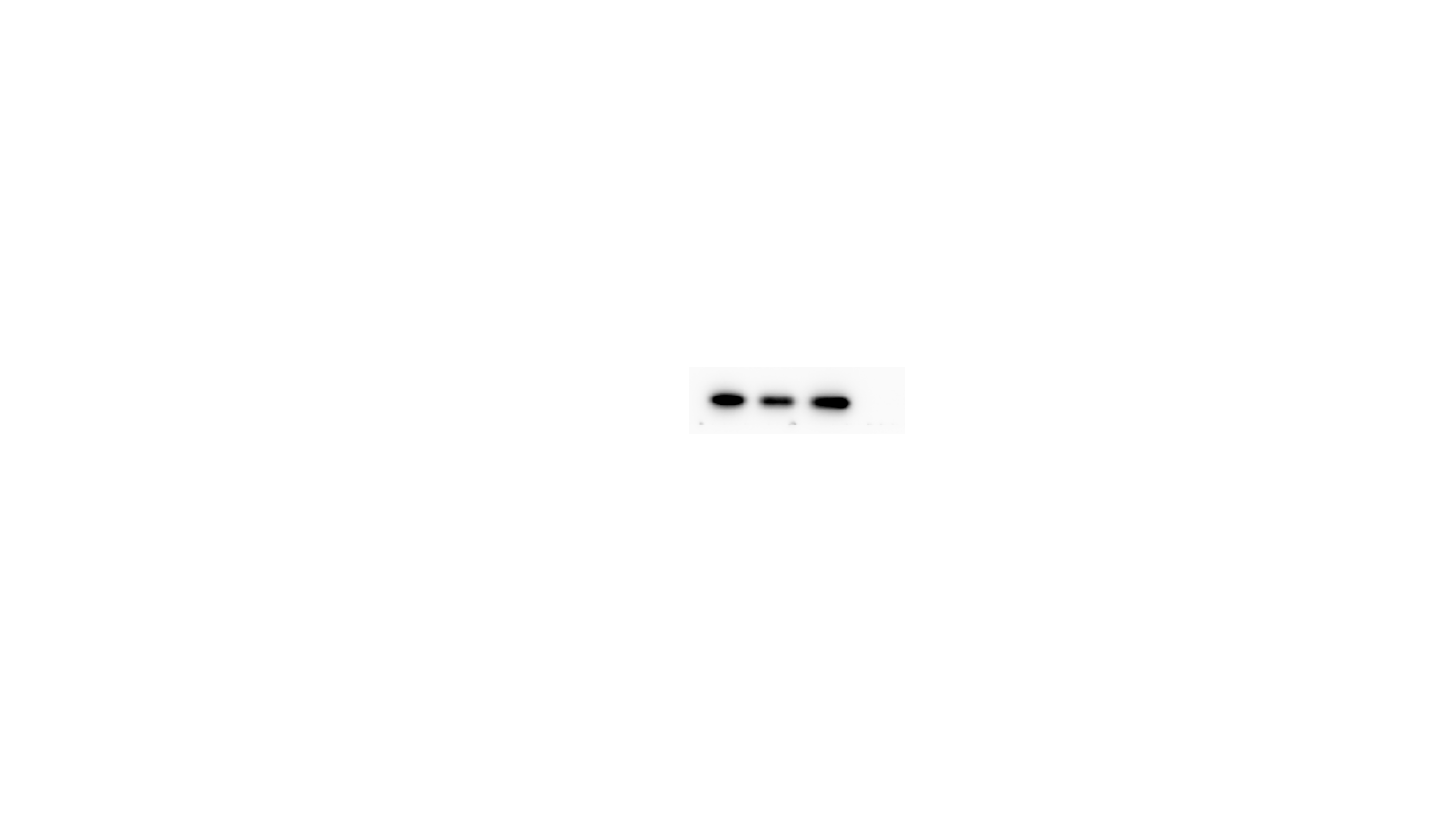

Supplement: Supplementary file 1 [file biomolecules-13-01298-s001.zip › WB original figure of beta-actin in TRIM6 knockdown group.tif]

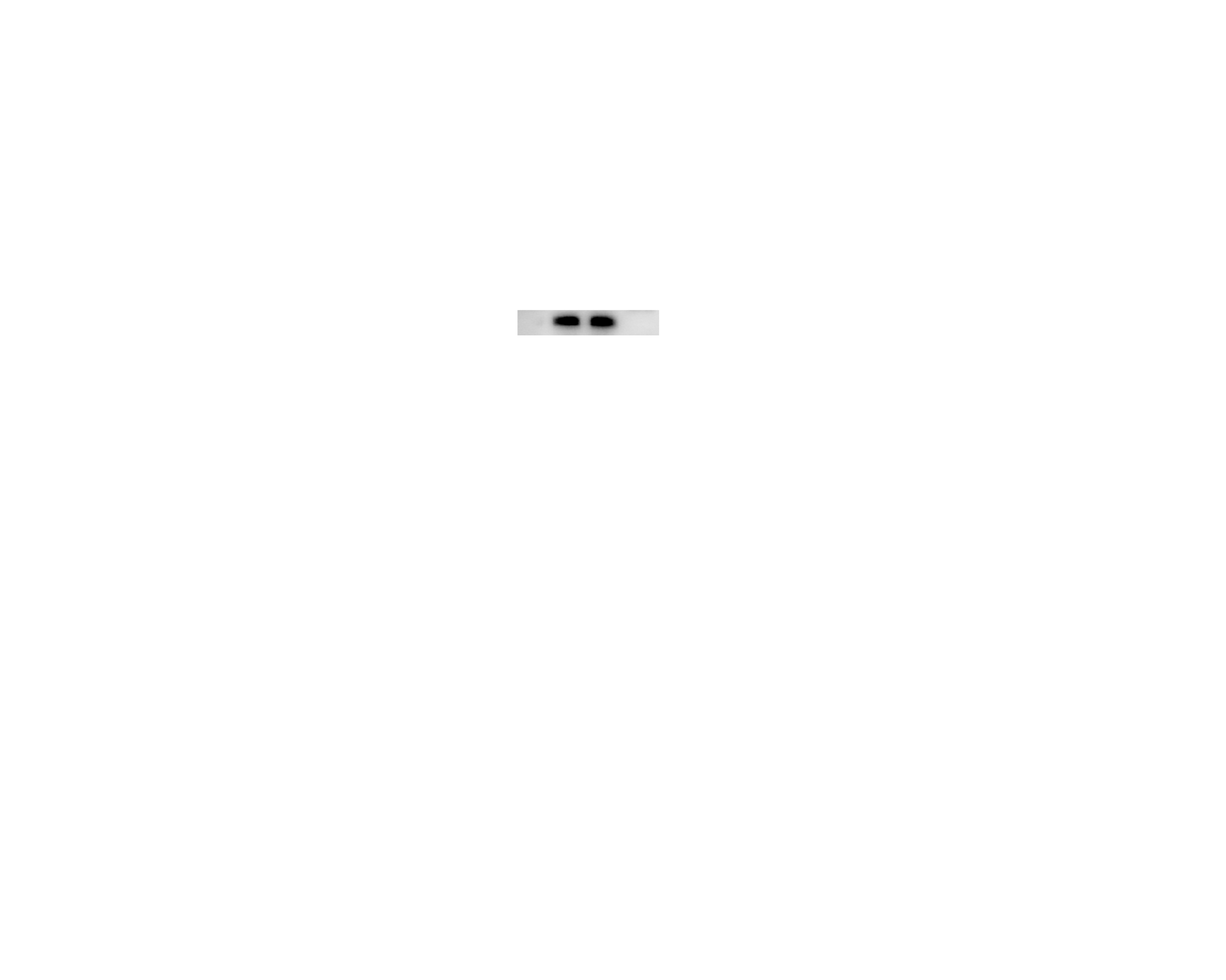

Supplement: Supplementary file 1 [file biomolecules-13-01298-s001.zip › WB original figure of beta-actin in TRIM6 overexpression group.tif]

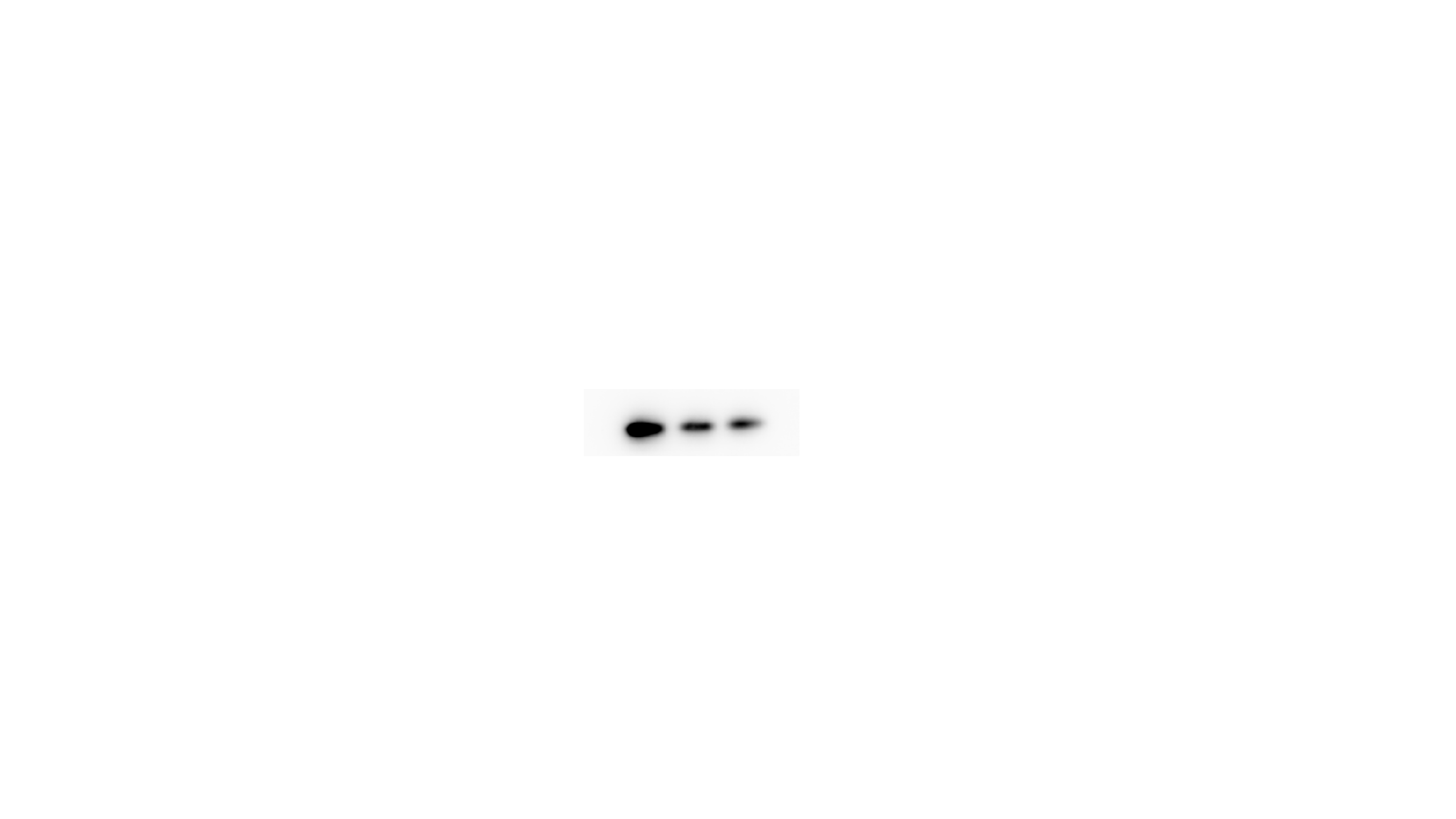

Supplement: Supplementary file 1 [file biomolecules-13-01298-s001.zip › WB original figure of TRIM6 in TRIM6 knockdown group.tif]

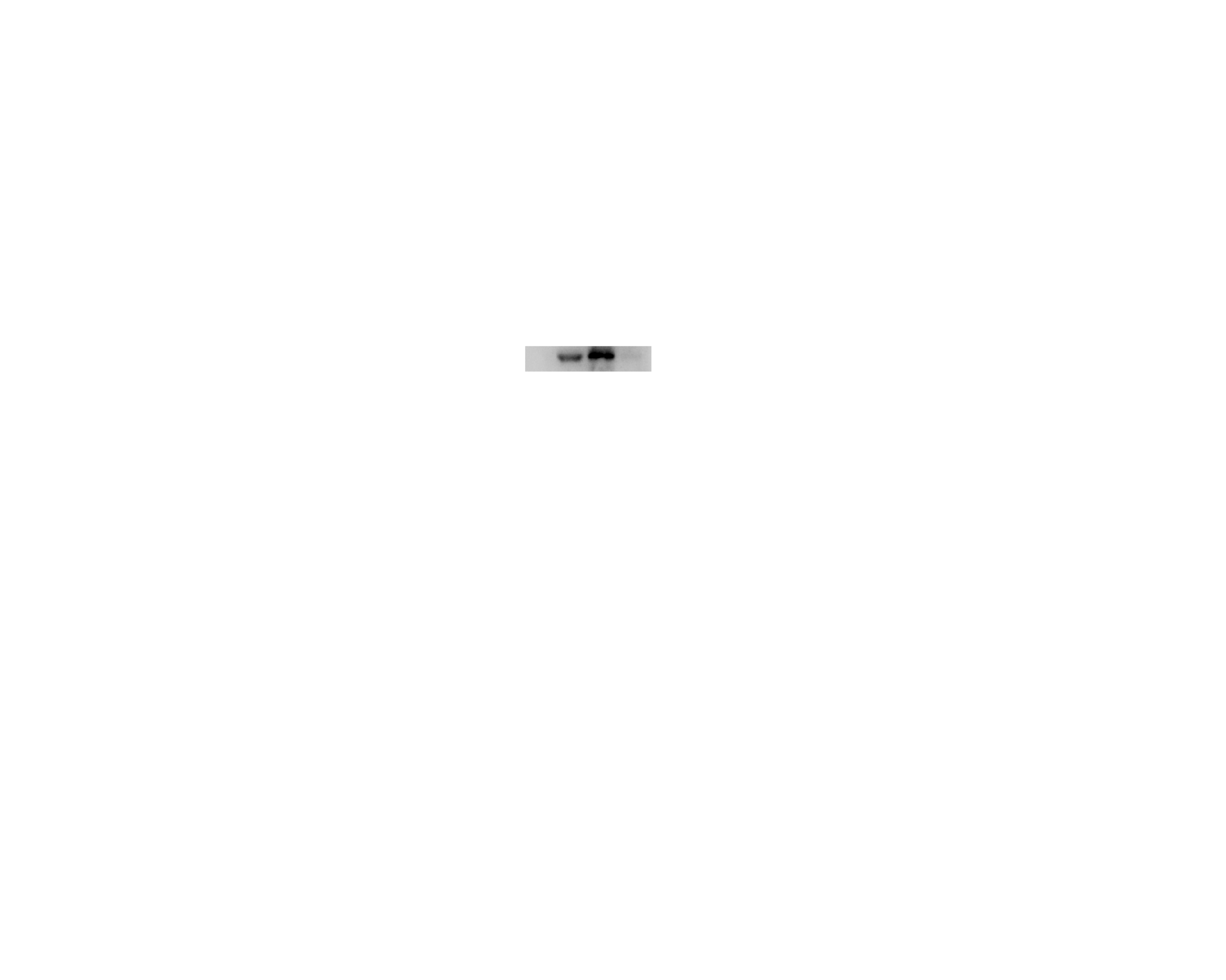

Supplement: Supplementary file 1 [file biomolecules-13-01298-s001.zip › WB original figure of TRIM6 in TRIM6 overexpression group.tif]
